# Future collapse: how optimistic should we be?

**DOI:** 10.1098/rspb.2013.1373

**Published:** 2013-09-22

**Authors:** Paul R. Ehrlich, Anne H. Ehrlich

**Affiliations:** Department of Biology, Stanford University, Stanford, CA, USA

Prof. Kelly FRS is optimistic about the chances of avoiding a collapse, but sadly we find his arguments entirely unpersuasive. For example, have Malthus (or we) really been wrong about food security? Roughly 850 million people are seriously undernourished (lacking sufficient calories) today, and perhaps 2 billion are malnourished (lacking one or more essential nutrients) [1]. When Malthus lived, there were only about 1 billion people on the planet. We agree that there are many things that *could* be done to feed today's population of 7.1 billion, or even perhaps over 9 billion in 2050. Many of them (e.g. limiting waste) have been discussed for 50 years with little sign of progress. We do not think any serious analyst doubts that, if it were equitably distributed, today's food production could nourish everyone adequately. Equally, we know of no serious analyst who believes such distribution is likely in the future. The concern is that climate disruption combined with other problems with the agricultural system will make it impossible to feed an ever larger future population, even if equal distribution were achieved. That concern is reinforced by the recent observation that, even before the likely heavy impacts of climate disruption on agriculture appear, production is failing to keep pace with projected needs [2].

There has been an important decline in birth rates in much of the world, but the median projections still foresee an increase of approximately 2.5 billion people by 2050 [3], and recently there have been worrying signs of ‘a fundamental change in the well-established negative relationship between fertility and development’ ([4], p. 741). Most recently, the UN has revised its projections of future population sizes *upward* [5], projecting a 2100 population of roughly 11 billion. On the brighter side, there is certainly good reason to think that, with modern contraception and communications, the population trajectory *could* be changed, leading to reduced fertility in rapidly growing populations if a significant international effort to promote women's rights and family planning in every nation were mounted.

Much of Prof. Kelly's criticism of our article is that our treatment of climate disruption, which we discussed first in 1968 [6] and many times subsequently [7–10] without significant dispute, is mistaken. This is critical, since, for example, the ability to feed people has been dependent on a stable climate, and most other elements of the human predicament have a climate component. We are not climate scientists, but follow the technical literature closely. So we chose to use the consensus view of the climate situation, which is accepted by more than 95% of those scientists, and the best and most experienced of them on the whole [11–14].

Those who would like a point-by-point scientific refutation of the positions cited by Kelly can consult the electronic supplementary material, which refers people to the SkepticalScience website (http://www.skepticalscience.com/argument.php). The website deals with each of the ‘myths’ from the standard ‘denier’ literature, which Kelly promotes. The site gives several levels of detail, provides references to the refereed scientific literature and has a well-moderated discussion of each view.

Of course, science never ‘proves’ anything, and consensus does not guarantee correctness. Despite the gigantic effort that has gone into studying the Earth's atmosphere, the entire climate science community could be mistaken. In this case, we hope Kelly has seen through evidence that has confused that community and so alarmed it, and if he is correct we certainly would have a brighter view of the future. But we saw no choice when commenting outside of our own areas of expertise but to give the climate scientists' near-unanimous view.

We think mobile phone technology does not speak well to the basic issues of overconsumption, especially since its environmental and socio-political consequences are hardly established. Pole-to-pole pollution with toxic chemicals (many of which are endocrine-disrupting compounds), destruction of biodiversity and decay of ecosystem services (none addressed by Kelly) are critical parts of the consumption problem and may be even more serious than climate disruption. We will stick with the references we cited, plus more recently the key point about resource depletion made by Davidson & Andrews [15].

Much of the rest of Kelly's criticism strikes us as proof by vigorous assertion, but we will leave that for others to decide. It seems to us that this is the wrong time in history for unsubstantiated optimism.

Finally, Kelly states, ‘The mainstream scientific and engineering community can see nothing that suggests an imminent collapse of civilization … ’. That's one phrase in Kelly's article with which we heartily agree, assuming that he does not consider diverse scientific signatories of earlier warning statements [16,17], climate scientists or ecologists ‘mainstream’, since they have spoken out clearly on the issue [18], most recently in very large numbers [19]. The lack of foresight Prof. Kelly notes in the engineering community is one of the main reasons we see the odds of collapse as greater than he does. We hope the complacency of that community is justified and the future is bright, but fear that it and Kelly are dead wrong.

## References

[1] FAO (2012). *The state of food insecurity in the world 2012*. Rome, Italy: Food and Agriculture Organization..

[2] Ray DK Mueller ND West PC Foley JA 2013 Yield trends are insufficient to double global crop production by 2050. PLoS ONE 8, e66428 (doi:10.1371/journal.pone.0066428) 23840465 10.1371/journal.pone.0066428 PMC3686737

[3] Population Reference Bureau 2012 2012 World Population Data Sheet. Washington, DC: Population Reference Bureau

[4] Myrskylä M Kohler H-P Billari FC 2009 Advances in development reverse fertility declines. Nature 460, 741–743 (doi:10.1038/nature08230) 19661915 10.1038/nature08230

[5] Engelman R. 2013 Our overcrowded planet: a failure of family planning See http://e360.yale.edu/feature/our_overcrowded_planet_a_failure_of_family_planning/2666

[6] Ehrlich PR 1968 The population bomb. New York, NY: Ballantine Books

[7] Ehrlich PR Ehrlich AH Holdren JP 1977 Ecoscience: population, resources, environment. San Francisco, CA: WH Freeman and Co

[8] Ehrlich PR Ehrlich AH 1990 The population explosion. New York, NY: Simon and Schuster

[9] Daily GC Ehrlich PR Mooney HA Ehrlich AH 1991 Greenhouse economics: learn before you leap. Ecol. Econ. 4, 1–10 (doi:10.1016/0921-8009(91)90002-V)

[10] Ehrlich PR Ehrlich AH 2009 The dominant animal: human evolution and the environment, 2nd edn Washington, DC: Island Press

[11] Oreskes N 2004 The scientific consensus on climate change. Science 306, 1686 (doi:10.1126/science.1103618) 15576594 10.1126/science.1103618

[12] Anderegg WRL Prall JW Harold J Schneider SH 2010 Expert credibility in climate change. Proc. Natl Acad. Sci. USA 107 12 107–12 109 (doi:10.1073/pnas.1003187107) 10.1073/pnas.1003187107 PMC2901439 20566872

[13] Bray D 2010 The scientific consensus of climate change revisited. Environ. Sci. Policy 13, 340–350 (doi:10.1016/j.envsci.2010.04.001)

[14] Cook J Nuccitelli D Green SA Mark R Winkler B Painting R Way R Jacobs P Skuce A 2013 Quantifying the consensus on anthropogenic global warming in the scientific literature. Environ. Res. Lett. 8, 024024 (doi:10.1088/1748-9326/8/2/024024)

[15] Davidson DJ Andrews J 2013 Not all about consumption. Science 339, 1286–1287 (doi:10.1126/science.1234205) 23493704 10.1126/science.1234205

[16] Union of Concerned Scientists 1993 World scientists’ warning to humanity. Cambridge, MA: Union of Concerned Scientists

[17] Nat ional Academy of Sciences 1993 Population summit of the world's scientific academies. Washington, DC: National Academies Press

[18] May RM 2006 Threats to tomorrow's world. Notes Rec. R. Soc. 60, 109–130 (doi:10.1098/rsnr.2005.0134)

[19] Barnosky AD 2013 Scientific consensus on maintaining humanity's life support systems in the 21st century: information for policy makers. See http://mahb.stanford.edu/consensus-statement-from-global-scientists

